# Where should the safe limits of alcohol consumption stand in light of liver enzyme abnormalities in alcohol consumers?

**DOI:** 10.1371/journal.pone.0188574

**Published:** 2017-12-05

**Authors:** Onni Niemelä, Markus Niemelä, Risto Bloigu, Mauri Aalto, Tiina Laatikainen

**Affiliations:** 1 Department of Laboratory Medicine and Medical Research Unit, Seinäjoki Central Hospital and University of Tampere, Seinäjoki, Finland; 2 Department of Medicine, University of Oulu, Oulu, Finland; 3 Medical Informatics and Statistics Research Group, University of Oulu, Oulu, Finland; 4 Department of Psychiatry, Seinäjoki Central Hospital and University of Tampere, Tampere, Finland; 5 National Institute for Health and Welfare (THL), Helsinki, Finland; 6 The Institute of Public Health and Clinical Nutrition, University of Eastern Finland, Kuopio, Finland; University Hospital Llandough, UNITED KINGDOM

## Abstract

**Objectives:**

To estimate the prevalence and risk factors for abnormal liver enzymes in a large age- and gender stratified population-based sample of apparently healthy individuals with or without alcohol consumption and other health-related risk factors (adiposity, physical inactivity, smoking).

**Methods:**

Data on alcohol use, smoking, diet and physical activity were recorded using structured questionnaires from 13,976 subjects (6513 men, 7463 women, aged 25–74 years) in the national FINRISK studies. Alcohol data was used to categorize the participants into abstainers, light drinkers, moderate drinkers and heavy drinkers. Serum gamma-glutamyltransferase (GGT) and alanine aminotransferase (ALT) activities were measured using standard kinetic methods.

**Results:**

Male light drinkers, moderate drinkers and heavy drinkers showed significantly higher relative risks of abnormal GGT than abstainers: 1.37 (95% confidence interval 1.11 to 1.71, p < 0.01), 2.72 (2.08 to 3.56, p < 0.0005), and 6.10 (4.55 to 7.17, p < 0.0005), respectively. Corresponding values for women were 1.22 (0.99 to 1.51, p = 0.065), 1.90 (1.44 to 2.51, p < 0.0005), and 5.91 (3.80 to 9.17, p < 0.0005). Estimated threshold doses for a significant GGT elevation was 14 standard weekly alcohol doses for men and 7 for women. Excess body weight and age over 40 years modulated the thresholds towards smaller quantities of alcohol. The risk of abnormal GGT was also significantly influenced by physical inactivity and smoking. The relative risks of abnormal ALT activities were increased in male heavy drinkers, especially in those presenting with adiposity and sedentary lifestyle.

**Conclusions:**

Alcohol use markedly increases the risk for abnormal liver enzyme activities in those presenting with age over 40 years, obesity, smoking or sedentary lifestyle. The data should be considered in public health recommendations and in the definitions of safe limits of alcohol use.

## Introduction

Alcohol consumption accounts for a significant proportion of life years lost to disability [1–3]. Based on scientific evidence about the harmful consequences of alcohol drinking, the definition of heavy or at-risk drinking has been rapidly evolving over the past two decades. The data gathered so far has also prompted revisions of governmental guidelines in several countries [2, 4–6]. In current societies approximately every sixth adult individual have been estimated to drink alcohol in amounts, which exceed the limits that are known to increase the risk of multiple medical problems (24 standard drinks of alcohol per week for men and 16 drinks for women) [1, 4, 5]. However, the health risks resulting from consumption of more modest amounts of alcohol and the issue of safe limits of alcohol use have remained as matters of long-standing controversy.

Recently, evidence has accumulated to indicate that even light to moderate alcohol drinking may increase the risk of certain cancers [6–8] and adverse brain outcomes in long-term follow-ups [9]. Studies have further indicated that the activities of common liver enzymes, ALT and GGT, may increase from baseline as a result of relatively low alcohol drinking levels especially in individuals with adiposity [10–13]. With epidemic levels of both obesity and excessive alcohol intake doctors are currently encountering increasing numbers of abnormal liver function tests which may indicate silent liver disease [2, 10, 14]. The early changes in the activities of liver enzymes in such patients may also predict both hepatic and extra-hepatic health risks, including metabolic syndrome, and cardio- or cerebrovascular events [15–17]. Changes in GGT activities appear to be mechanistically linked with the activation of oxidative stress [18–20], whereas serum ALT rather marks disturbed liver cell integrity or cellular adaptation to maintain energy homeostasis [21, 22].

Recent advances in individualized medicine have also emphasized a more systematic use of biomarker data for the assessment of lifestyle and dietary interventions aimed at reducing disorders caused by hazardous drinking or excess body weight [1, 10, 23]. A more widespread use of laboratory tests has, however, been hampered by the lack of uniform definitions of biomarker normal ranges for even the most commonly used biomarkers, such as liver enzymes, and lack of knowledge on the safe limits of alcohol intake [24, 25]. There is also a paucity of studies observing the relationships between mild to moderate levels of alcohol drinking and the early-phase changes in liver enzymes in large populations stratified by key covariates [10, 25].

In the present study we collected data from a large national FINRISK population health survey to determine the relative risks of abnormal liver enzyme findings in apparently healthy individuals with detailed records on alcohol consumption, diet and other health-related behaviour, including physical activity. The recently established ULNs for liver enzymes based on abstainers with normal body weight were used as reference [11]. The purpose of the study was to offer new perspectives on the relationships between alcohol use, adiposity, sedentary life style and liver enzyme abnormalities, which would have important implications for public health.

## Materials and methods

### Participants

Data was collected from a cross-sectional population health survey (The National FINRISK study) carried out in six different geographic areas in Finland in years 1997, 2002, and 2007. This survey was originally set out to examine risk factors of chronic, non-communicable diseases and modifiable behavioural risk factors. For this purpose, an age- and gender stratified random sample was drawn from the population register according to an international WHO MONICA (Monitoring trends and determinants in cardiovascular disease) protocol [26]. The clinical examinations included physical measurements, laboratory tests and detailed questionnaires encompassing alcohol intake, current health status, diet, smoking, current physical activity, medical history and socioeconomic factors [26–28]. Body weight and height were measured to the nearest 0.1 kg and 0.1 cm, respectively. Body mass index (BMI, kg/m^2^) was calculated as a measure of relative body weight. Waist circumference was measured to the nearest 0.5 cm between the lowest rib and the iliac crest while the subject was at minimal respiration.

The subjects included were devoid of any clinical signs of liver disease, ischaemic heart or brain disease, diabetes or abnormal glucose tolerance test, hypertension or active infection at the time of the study. The final study population consisted of 13,976 apparently healthy individuals: 6513 men and 7463 women (mean age 45 ± 13 years, range 25–74 years) who completed the questionnaires and attended the medical examinations. The response rates in the 1997, 2002 and 2007 surveys were 73.4%, 71.4% and 66.9%, respectively. Alcohol consumption was assessed with structured questionnaires including information on the type of beverage consumed and the quantity and frequency of consumption using a retrospective recall method registering consumption from the past weeks and one year prior to blood sampling [28, 29]. The amount of ethanol in different beverages was quantitated based on defined portion sizes as follows: regular beer 12 grams (1/3 L), strong beer 15.5 grams (1/3 L), long drink 15.5 grams (1/3 L), spirit 12 grams (4 cL), wine 12 grams (12 cL) and cider 12 grams (1/3 L). A dose of 12 grams of pure ethanol was considered as one standard drink.

The data on alcohol consumption was subsequently used to categorize the population by gender and drinking habits as follows: 1. persons who reported no current alcohol consumption were referred to as non-drinkers (abstainers), 2. light drinkers consumed between 1–13 drinks (men) or 1–6 drinks (women), 3. moderate drinkers consumed 14–23 drinks (men) or 7–15 drinks (women) and 4. heavy drinkers consumed more than 23 drinks (men) or more than 15 drinks (women) per week. In the assessment of relative risks of abnormal liver enzyme activities in more detail the category of light drinkers was further separated into subgroups of very light drinkers: men < 7 (n = 2166), women < 3.5 (n = 2092) drinks per week and light drinkers men ≥ 7 and < 14 (n = 1436), women ≥ 3.5 and < 7 (n = 1200) drinks per week.

Smoking and coffee consumption were assessed with a set of standardized questions and expressed as the amount of cigarettes per day and as the intake of standard servings of coffee (cups) per day, respectively. Physical activity and the number and total time used for physical exercises were registered using structured questionnaires [28, 30]. The data was used to classify the population into the subgroups of 1. moderate or vigorous activity (over 4 hours of activity per week including brisk running, walking, cross-country skiing, swimming or other strenuous exercises) 2. light (0.5–4 hours per week including walking, cycling, gardening or other moderate activities), and 3. sedentary activity (less than 0.5 hours per week).

All surveys were conducted in accordance with the Declaration of Helsinki according to the ethical rules of the National Public Health Institute. Written informed consent was obtained from all participants. The approval for this study was received from the Coordinating Ethics Committee of the Helsinki Hospital District.

### Laboratory analyses

Serum ALT and GGT were measured by standard kinetic methods following recommendations of the European Committee for Clinical Laboratory Standards (ECCLS) on an Abbott Architect clinical chemistry analyser (Abbott Laboratories, Abbott Park, IL, USA).

### Statistical methods

Values are expressed as mean ± SD or mean ± 95% confidence interval (CI). Comparisons in the distribution of the data were carried out with a chi square test for trend and pr-test on the equality of proportions using Stata statistical data analysis software (StataCorp LP, TX, USA). For comparisons between groups, Student’s t-test or ANOVA with Tukey’s HSD as a post hoc test for multiple factors were used. ANOVA with Dunnett post hoc test was used to determine the threshold levels of alcohol doses for initiating a significant elevation in liver enzymes based on the data with a separate category for each weekly standard drink. Logistic regression was used to assess whether drinking status and the covariates associated with the abnormal liver enzyme levels. Both univariable and multivariable analyses were performed and the results of multivariable analyses are reported. In the analyses the participants with missing data were listwise excluded. The analyses were carried out with IBM SPSS Statistics 22.0 (Armonk, NY: IBM Corp.). A p-value < 0.05 was considered statistically significant.

## Results

The main demographic characteristics and lifestyle factors of the participants classified to subgroups according to alcohol consumption and gender are summarized in Table 1. Among men, 26.0% of the population were abstainers, 55.3% were light drinkers, 12.0% were moderate drinkers and 6.7% were heavy drinkers. In women the corresponding percentages were 41.2%, 44.1%, 12.7%, and 2.0%.

**Table 1:** Main characteristics of the study population, as classified according to drinking status.

Men		Abstainers	Light drinkers	Moderate drinkers	Heavy drinkers
	Alcohol consumption	0 drinks/week	1–13 drinks/week	14–23 drinks/week	≥ 24 drinks/week
	N (%)	1696 (26.0)	3602 (55.3)	780 (12.0)	435 (6.7)
	Age, years, mean ± SD	46.1 ± 13.8 (n = 1696)	44.8 ± 12.9 (n = 3602)	44.0 ± 11.9 (n = 780)	44.8 ± 11.3 (n = 435)
	BMI	26.3 ± 3.8 (n = 1554)	26.2 ± 3.5 (n = 3279)	26.6 ± 3.9 (n = 699)	26.9 ± 4.4 (n = 382)
	Waist circumference, cm	92.8 ± 10.9 (n = 1554)	92.6 ± 10.2 (n = 3267)	94.2 ± 11.2 (n = 696)	96.3 ± 12.4 (n = 380)
	Smoking, cigarettes/day	4.3 ± 8.8 (n = 1685)	4.4 ± 8.1 (n = 3562)	8.0 ± 10.2 (n = 773)	11.4 ± 12.6 (n = 426)
	Coffee, cups/day	4.8 ± 3.6 (n = 1670)	4.7 ± 3.1 (n = 3571)	5.1 ± 3.4 (n = 780)	4.9 ± 4.0 (n = 430)
	Physical activity, number of exercises/week	2.5 ± 2.2 (n = 600)	2.3 ± 1.9 (n = 1366)	2.0 ± 2.1 (n = 328)	2.0 ± 2.3 (n = 166)
Women		Abstainers	Light drinkers	Moderate drinkers	Heavy drinkers
	Alcohol consumption	0 drinks/week	1–6 drinks/week	7–15 drinks/week	≥ 16 drinks/week
	N (%)	3072 (41.2)	3292 (44.1)	947 (12.7)	152 (2.0)
	Age, years, mean ± SD	44.3 ± 13.5 (n = 3072)	43.8 **±** 12.2 (n = 3292)	42.4 **±** 11.4 (n = 947)	45.4 **±** 10.8 (n = 152)
	BMI	25.8 **±** 4.8 (n = 2818)	25.1 **±** 4.4 (n = 3022)	24.9 **±** 4.0 (n = 851)	25.6 **±** 4.1 (n = 125)
	Waist circumference, cm	82.0 **±** 12.1 (n = 2722)	80.5 **±** 11.3 (n = 3010)	80.6 **±** 10.9 (n = 849)	83.6 **±** 10.8 (n = 125)
	Smoking, cigarettes/day	2.0 **±** 5.3 (n = 3059)	2.1 **±** 5.2 (n = 3274)	3.9 **±** 6.4 (n = 937)	8.7 **±** 9.0 (n = 149)
	Coffee, cups/day	3.7 **±** 2.6 (n = 3038)	3.7 **±** 2.5 (n = 3281)	3.8 **±** 2.5 (n = 940)	3.8 **±** 2.4 (n = 152)
	Physical activity, number of exercises/week	2.5 **±** 2.1 (n = 1146)	2.6 **±** 2.1 (n = 1300)	2.3 **±** 1.9 (n = 394)	2.7 **±** 2.4 (n = 62)

BMI, body mass index

The upper normal limits used for ALT and GGT were based on previously described values defined by calculating 97.5^th^ percentiles of the data based on normal weight non-drinkers [11]. The amount of alcohol (mean ± SD) consumed in those exceeding the ULNs in GGT analyses in the present population was 170 ± 206 grams per week for men and 55 ± 78 grams per week for women (Table 2). For ALT, the corresponding levels were 144 ± 194 grams per week for men and 42 ± 63 grams per week for women (Table 2).

**Table 2:** Mean alcohol consumption (grams per week) in groups according to laboratory test ULN.

		< ULN	≥ ULN	
		mean ± SD	mean ± SD	p
GGT				
	Men	82.1 ± 110.1	170.3 ± 206.3	< 0.0005
	Women	33.7 ± 50.8	54.6 ± 78.4	< 0.0005
ALT				
	Men	91.1 ± 122.4	144.0 ± 193.9	< 0.0005
	Women	35.1 ± 50.4	41.8 ± 62.9	0.160

GGT, gamma-glutamyltransferase; ALT, alanine aminotransferase; ULN, upper limit of normal

**Fig 1:**
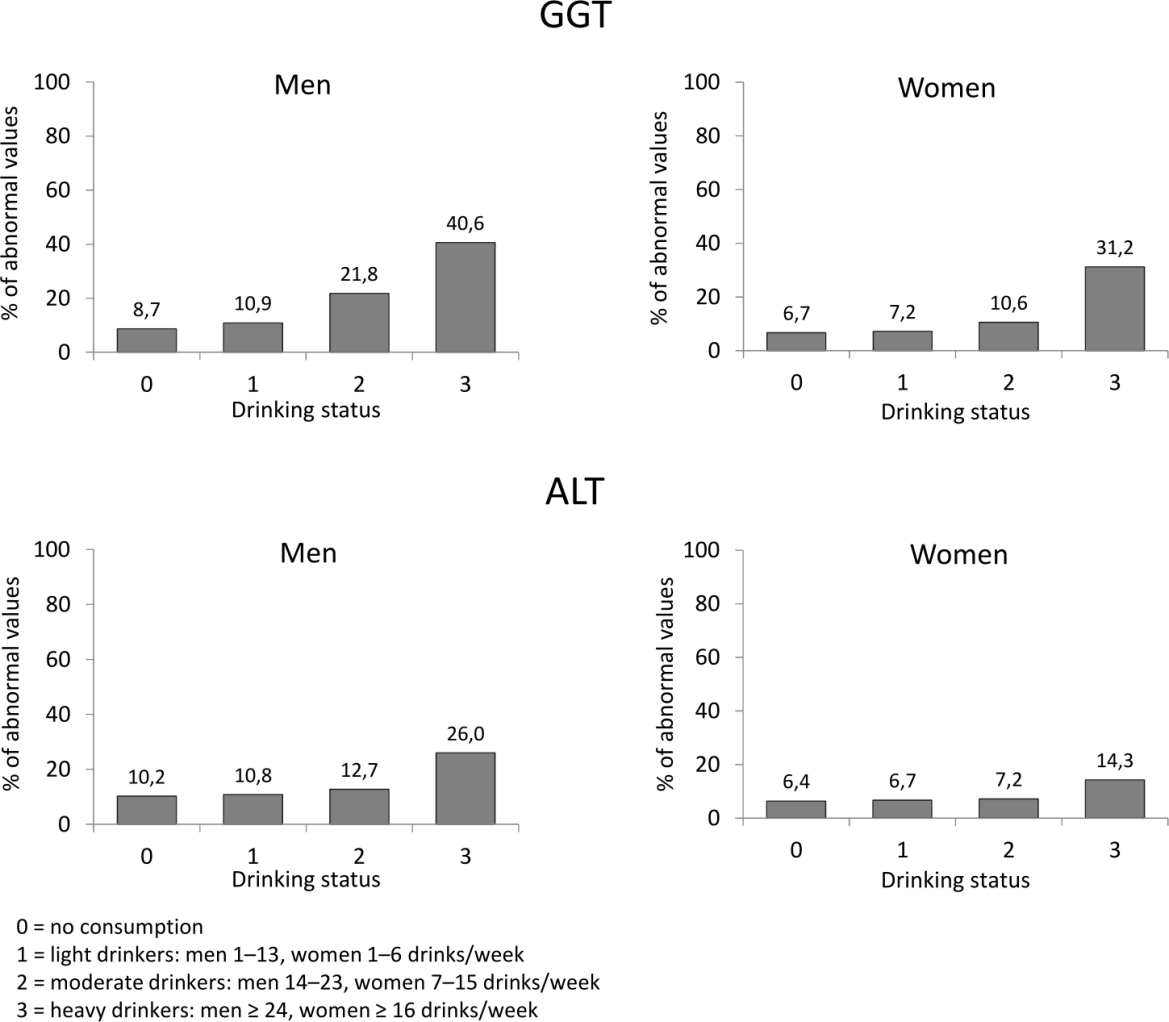
Incidence of abnormal GGT and ALT activities in groups classified according to drinking status.

Fig 1 shows the frequencies of abnormal liver enzymes from the different subgroups classified according to drinking status. In men, abnormal GGT findings were significantly more frequent in light drinkers (10.9%) (p < 0.01), moderate drinkers (21.8%) (p < 0.001) and heavy drinkers (40.6%) (p < 0.001) than in abstainers (8.7%). In women, the likelihood of abnormal GGT findings was increased in moderate drinkers (10.6%) (p < 0.001) and in heavy drinkers (31.2%) (p < 0.001) when compared to abstainers (6.7%) or light drinkers (7.2%) (p < 0.001 for each comparison). For ALT, the group of heavy drinkers showed abnormal values more frequently than the group of abstainers (p < 0.001 for men, p < 0.01 for women) (Fig 1).

In the analyses of the incidences of abnormal liver enzyme values based on alcohol consumption reported from the past 12 months, the estimates of weekly alcohol consumption were found to be in good agreement with the estimates obtained from the data on more recent drinking (r = 0.664, p < 0.0001). The incidences of elevated liver enzymes were, however, higher in the subgroups classified as heavy drinkers based on the latter approach (Fig 1) than in the corresponding subgroups formed based on 12-mo data (GGT: 31.4% men, 26.5% women; ALT 21.1% men, 12.8% women). No significant differences occurred in the corresponding comparisons of abstainers, light drinkers or moderate drinkers. The individuals who could be classified as former drinkers (n = 314, 159 men, 155 women) reporting previous alcohol consumption but no alcohol consumption from the past 12 months showed low rates of elevated liver enzyme values (GGT: 7.1% men, 2.4% women; ALT 6.0% men, 1.4% women).

**Fig 2:**
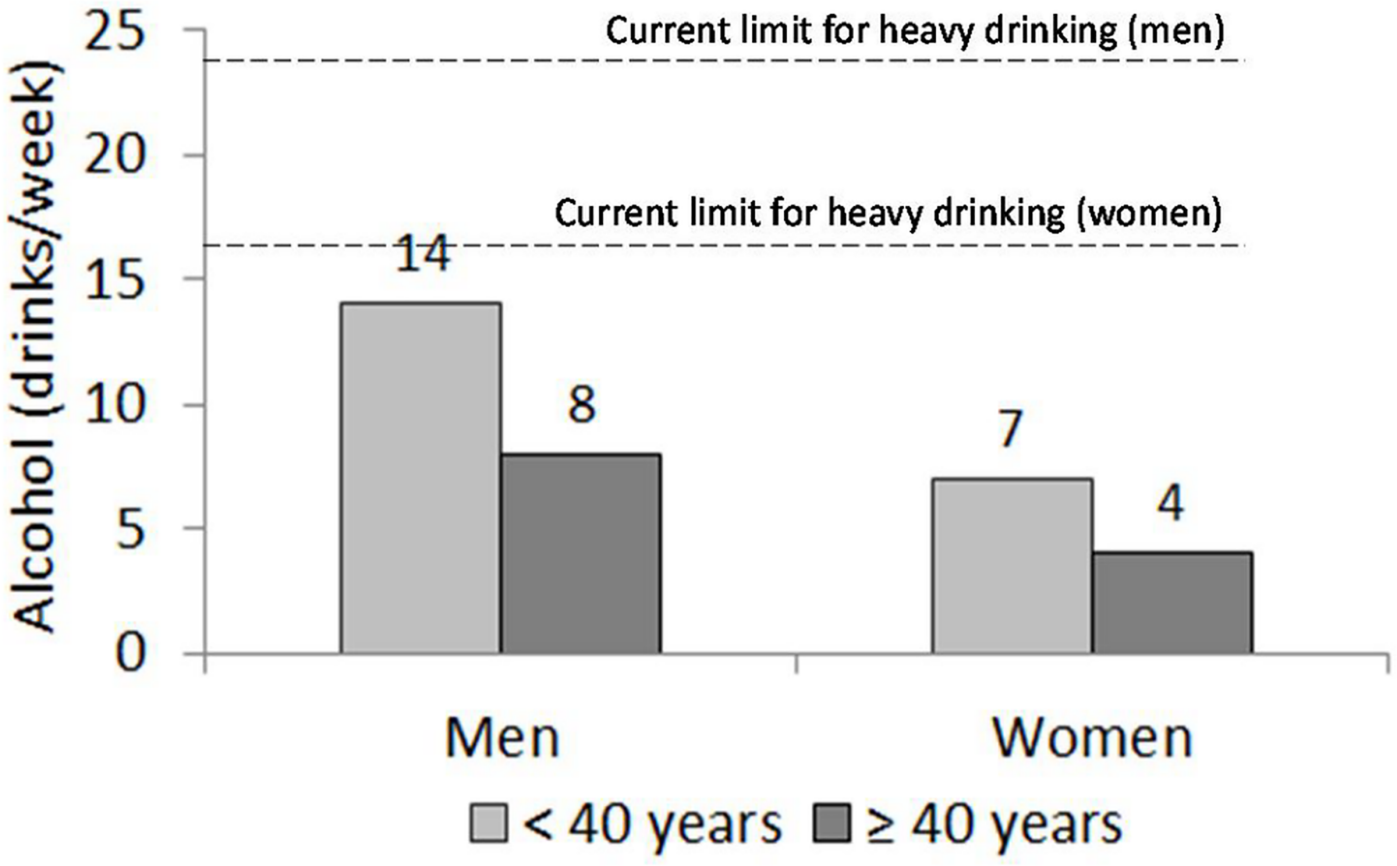
Estimated threshold doses of alcohol consumption (standard drinks /week) for initiating a significant GGT activation. The levels leading to GGT increases especially in those above 40 years of age are markedly lower than the current limits of heavy drinking in many Western countries (men: 24 drinks, women: 16 drinks) (dashed line).

**Fig 3:**
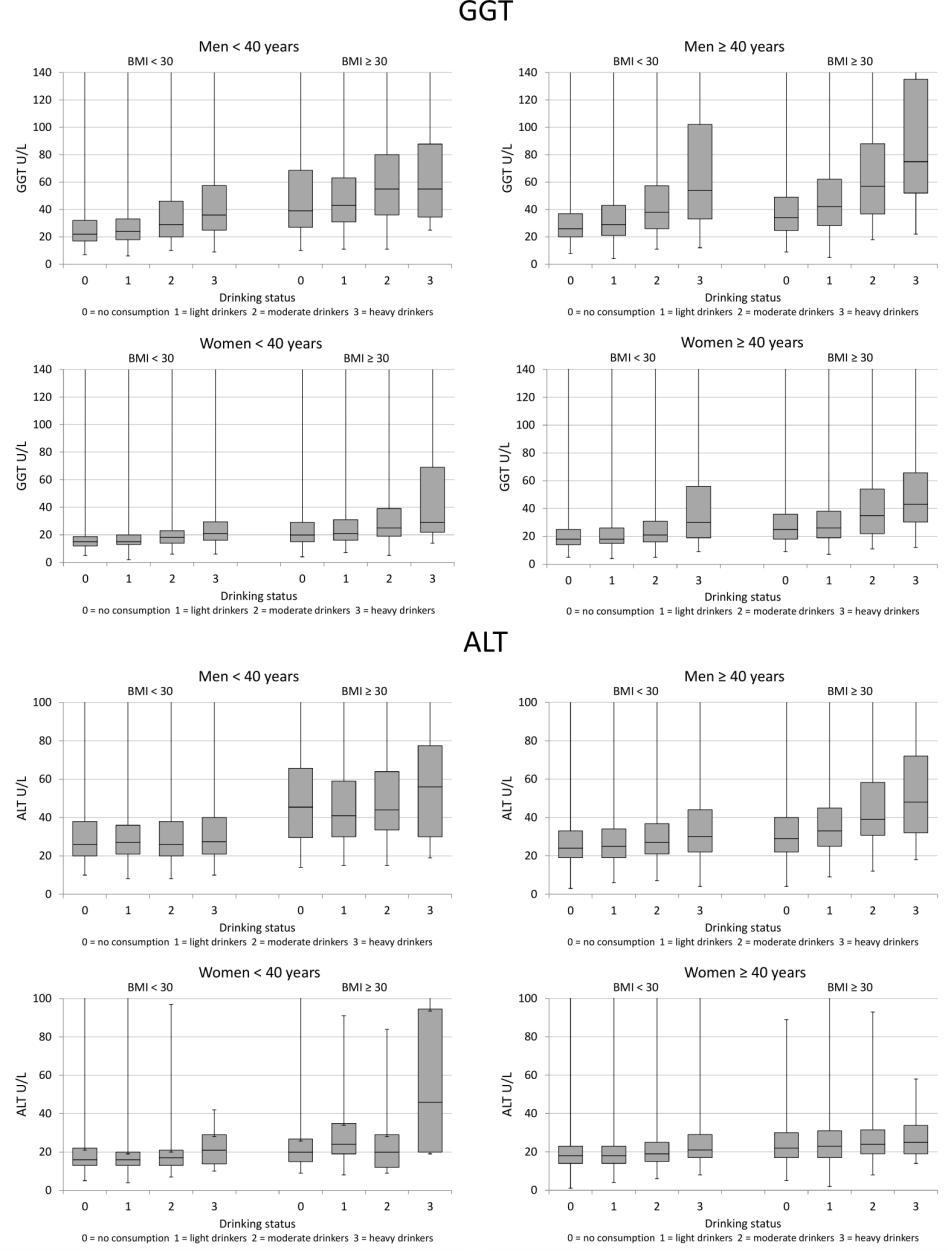
Serum GGT and ALT in subgroups classified according to drinking status, age and BMI. Data are shown as median and interquartile ranges.

The estimated threshold alcohol doses for initiating a significant elevation in GGT activities were 14 standard drinks of weekly alcohol consumption for men and 7 drinks for women (Fig 2). Excess body weight and increasing age were found to modulate the thresholds towards smaller quantities (Fig 3).

Table 3 summarizes the multivariable relative risks of abnormal GGT and ALT findings according to drinking habits, age, waist circumference, physical activity and smoking. When compared with abstainers, male **l**ight drinkers, moderate drinkers and heavy drinkers had relative risks of abnormal GGT activities of 1.37 (95% confidence interval 1.11 to 1.71, p < 0.01), 2.72 (2.08 to 3.56, p < 0.0005), and 6.10 (4.55 to 7.17, p < 0.0005), respectively. Corresponding values for women were 1.22 (0.99 to 1.51, p = 0.065), 1.90 (1.44 to 2.51, p < 0.0005), and 5.91 (3.80 to 9.17, p < 0.0005). When the category of light drinkers was further separated into subgroups of very light drinkers (men < 7, women < 3.5 drinks per week) and light drinkers (men ≥ 7 and < 14, women ≥ 3.5 and < 7 drinks per week) the relative risks for abnormal activities were not found to be increased in any of the comparisons among very light drinkers (data not shown).

**Table 3:** Relative risks of abnormal liver enzyme levels in study subgroups from a cross-sectional FINRISK population survey.

		GGT					ALT			
		Men		Women		Men		Women	
		Multivariable relative risk	p	Multivariable relative risk	p	Multivariable relative risk	p	Multivariable relative risk	p
Drinking status									
	Abstainers								
	Light drinkers	1.37 (1.11 to 1.71)	0.004	1.22 (0.99 to 1.51)	0.065	1.09 (0.78 to 1.53)	0.614	1.08 (0.76 to 1.52)	0.666
	Moderate drinkers	2.72 (2.08 to 3.56)	< 0.0005	1.90 (1.44 to 2.51)	< 0.0005	1.19 (0.74 to 1.90)	0.481	1.27 (0.78 to 2.07)	0.344
	Heavy drinkers	6.10 (4.55 to 8.17)	< 0.0005	5.91 (3.80 to 9.17)	< 0.0005	3.10 (1.86 to 5.15)	< 0.0005	2.23 (0.94 to 5.33)	0.070
Age groups									
	< 40 yrs								
	40–49 yrs	1.27 (1.03 to 1.57)	0.026	1.75 (1.34 to 2.29)	< 0.0005	0.91 (0.66 to 1.26)	0.563	0.96 (0.63 to 1.45)	0.838
	50–64 yrs	1.23 (1.00 to 1.51)	0.051	2.70 (2.10 to 3.47)	< 0.0005	0.35 (0.24 to 0.51)	< 0.0005	1.36 (0.93 to 1.97)	0.109
	> 64 yrs	0.65 (0.46 to 0.93)	0.019	2.83 (1.99 to 4.02)	< 0.0005	0.17 (0.07 to 0.44)	< 0.0005	0.39 (0.14 to 1.10)	0.074
Waist circumference									
	Low risk *								
	High risk **	2.67 (2.19 to 3.27)	< 0.0005	1.54 (1.20 to 1.97)	0.001	3.08 (2.19 to 4.34)	< 0.0005	1.69 (1.12 to 2.54)	0.012
	Very high risk ***	5.07 (4.12 to 6.24)	< 0.0005	3.07 (2.45 to 3.84)	< 0.0005	6.00 (4.26 to 8.46)	< 0.0005	3.28 (2.25 to 4.78)	< 0.0005
Physical activity									
	Moderate/vigorous (reference)							
	Light	1.37 (1.11 to 1.69)	0.004	1.46 (1.12 to 1.89)	0.005	1.49 (1.03 to 2.15)	0.033	0.85 (0.58 to 1.25)	0.406
	Sedentary	1.51 (1.18 to 1.93)	0.001	1.55 (1.15 to 2.10)	0.004	1.96 (1.31 to 2.93)	0.001	0.83 (0.51 to 1.33)	0.437
Smoking									
	No smoking								
	1–9 cigarettes/day	1.03 (0.74 to 1.44)	0.869	1.26 (0.90 to 1.77)	0.179	1.26 (0.78 to 2.03)	0.350	1.02 (0.59 to 1.77)	0.954
	10–19 cigarettes/day	1.24 (0.97 to 1.58)	0.093	1.22 (0.91 to 1.65)	0.189	1.10 (0.73 to 1.65)	0.651	0.69 (0.40 to 1.19)	0.183
	20–29 cigarettes/day	1.61 (1.26 to 2.05)	< 0.0005	1.12 (0.70 to 1.79)	0.635	0.79 (0.51 to 1.24)	0.303	0.40 (0.12 to 1.30)	0.128
	≥ 30 cigarettes/day	1.56 (1.06 to 2.30)	0.024	2.05 (0.87 to 4.86)	0.102	0.81 (0.40 to 1.63)	0.549	4.14 (1.01 to 16.92)	0.048

GGT, gamma-glutamyltransferase; ALT, alanine aminotransferase

*, men < 94 cm, women < 80 cm

**, men 94–102 cm, women 80–88 cm

***, men > 102 cm, women > 88 cm

The risk of abnormal GGT was found to be significantly influenced by age, adiposity, physical activity and smoking (Table 3). For ALT, the relative risks of abnormal activities were increased only in male heavy drinkers. Adiposity and sedentary lifestyle also increased the likelihood of abnormal ALT levels (Table 3). The individuals with low or sedentary physical activity showed markedly higher relative risks for elevated liver enzymes than those engaged in physically demanding activities for at least 3 hours per week.

The analysis of the associations between coffee consumption and the risk for elevated liver enzyme values showed that GGT activities in those classified as heavy drinkers (whereas not in other drinking categories) were also significantly influenced by coffee drinking. Heavy drinkers consuming ≥ 4 cups of coffee showed significantly lower GGT levels (men 65 ± 74 U/L, women 41 ± 76 U/L) than the corresponding groups consuming 1–3 cups (men 91 ± 90 U/L, women 62 ± 120 U/L) or no coffee (men 120 ± 171 U/L, women 76 ± 95 U/L) (p < 0.001 for comparisons in both genders).

## Discussion

In this large cross-sectional population-based sample of apparently healthy individuals, we showed that the relative risk of abnormal liver enzyme activities is increased in individuals consuming alcohol in amounts considered as light to moderate drinking levels. In light of recent findings indicating that the early changes in the activities of GGT and ALT should also be regarded as important prognostic markers of both hepatic and extra-hepatic health risks [17, 19, 22], it may be assumed that the risk categories of alcohol drinking and associated individual health-related behaviour need further attention.

The large number of well-characterized study subjects offered us with the possibility to perform a comprehensive analysis of the relative risks of abnormal biomarker findings across all levels of alcohol consumption and other factors of lifestyle. Our study relied largely on the data obtained from common liver enzymes, ALT and GGT, both of which are readily induced by excessive alcohol consumption and are associated with relevant clinical outcomes. These liver enzymes may also be considered biomarkers of choice since they are easily measurable, cost-effective and easy to interpret by clinicians. Although current progress in clinical laboratories and quality control regimes has led to more consistent measurement procedures, several practical problems have remained unresolved including definitions of universal normal ranges. The ULNs for even the most commonly used biomarkers for liver status, ALT and GGT, currently show significant variation between individual laboratories and geographic areas [11, 25, 31, 32]. Thus, there is a continuing need for additional biomarker validation to translate the numerical values into clinically meaningful information, which would also allow more efficient international comparisons of the data.

Over the past decades reduction of harmful alcohol drinking has been an important target for public health policies. Although heavy alcohol use is known to be causally linked to over 60 distinct diseases, the association between light to moderate drinking and the overall risk of health problems has remained unclear [1, 5]. The concepts of beneficial, safe or harmful levels of ethanol intake have also remained as topics of great interest and lively debate. While other studies have suggested that light to moderate drinking is associated with beneficial health effects especially in those following a Mediterranean diet [33], other groups of investigators have not reached similar conclusions [34–36]. The questions on the dose-effect relationships between ethanol intake, biomarker changes and possible tissue injury have also remained unresolved. The present data supports the view that biochemical responses in the sequence of events leading to health problems may be expected to occur even in association with light to moderate drinking levels especially in those with precipitating risk factors. Recent findings by other groups of investigators have also emphasized the view that light to moderate drinking could be associated with an elevated risk of cancer [6–8], atrial fibrillation [37], left ventricular diastolic dysfunction [38], adverse brain outcomes [9] and an increase in all-cause mortality [39]. Although reductions in alcohol-attributable disease burden may obviously be expected to be achieved through a more efficient identification of high-risk individuals, alcohol-consuming patients seem rarely receive specific intervention in current clinical practice unless the situation is complicated by comorbidity.

The present data indicates that the influence of ethanol on liver enzymes is significantly driven by factors such as gender, age, excess body weight, physical inactivity or smoking, which should all be taken into account when discussing individually on the most appropriate levels of drinking. Although the primary mechanisms underlying such observations remain unknown at this time it is possible that all these conditions stimulate oxidative stress in an additive, gender- and age-dependent manner. GGT enzyme plays a pivotal role in the metabolism of glutathione (GSH), and elevated activities could sign a need to maintain intracellular GSH levels during oxidative stress [11, 15, 40–44]. While alcohol use and related harm are more prevalent in men, women seem to show elevated liver enzyme activities following consumption of smaller amounts of alcohol. In accordance with previous findings, alcohol consumption in those above 40 years of age appears to pose a higher risk towards elevated GGT levels [23], which could indicate a greater sensitivity towards oxidative stress upon aging or more prolonged alcohol exposure in such individuals [41, 45]. Unfortunately, quantitative data on lifetime exposure to ethanol was not available in this survey to test this hypothesis.

The presence of adiposity was also found to be an important driving force for abnormal liver enzymes. There may also be additive effects of excess body weight and alcohol use [46–48]. Recent studies have shown that the risk of non-alcoholic fatty liver disease (NAFLD) increases with both increasing levels of average daily alcohol consumption and increasing BMI [49]. The adverse effects of ethanol on the liver are also aggravated by high-fat-diets in experimental animal models [50]. Since in real life situations alcohol use often co-exists with obesity, synergistic health problems and metabolic comorbidities due to these two triggers may be expected to occur in an ever-increasing manner together with an increasing occurrence of abnormal liver function tests in the affected individuals [12, 13, 47, 51, 52]. Lifestyle and dietary therapies play a pivotal role in reversing the course of such conditions and consequently, there may be an increasingly important role of liver enzymes in monitoring treatment based on behavior change. There may also be emerging new applications for these enzymes as overall health indicators including health problems outside the liver [19]. Increases in ALT and GGT levels have been shown to predict deposition of triglycerides and fat in tissues and to increase extra-hepatic health risks, such as type 2 diabetes, metabolic syndrome, insulin resistance and cardio- or cerebrovascular events [15, 17, 19, 52–58]. Due to the ability of GGT to trigger iron-dependent oxidation of LDL in coronary plaques, studies have also proposed a role for GGT as a mechanistic link between fatty liver and atherosclerosis [55].

Physical inactivity is an increasingly common contributor to poor health across the world [59, 60]. Here physical inactivity and sedentary behaviour were found to markedly increase the risk for elevated liver enzymes supporting also the view that improvement of metabolic health through physical exercise could serve as a therapeutic approach to provide protection from the harmful consequences of alcohol and diet-induced adverse metabolic and obesogenic effects. The individuals engaged in physically demanding activities for at least 3 hours per week showed markedly lower risks for elevated liver enzymes than those with low or sedentary activity. It remains to be established whether physical exercise could also confer long-term benefits to hepatic health and alcohol-attributable disease burden in general [61–63].

In accordance with recent data by other groups of investigators [64] the present observations also point to a significant synergistic effect of smoking and alcohol use in increasing GGT levels. Smoking was more common in heavy drinkers of alcohol and it appears that the combination of heavy drinking and smoking also leads to additive hepatotoxic effects, especially in men [64, 65]. In addition, coffee consumption was found to interact with alcohol consumption and GGT levels such that a high intake of coffee in heavy alcohol drinkers was more likely to be associated with lower GGT levels than those in the corresponding group of heavy drinkers consuming no coffee. This observation points to a possible protective effect of coffee drinking towards alcohol-induced liver damage [66, 67]. However, in the present material the amount of coffee intake required for significant effects in GGT activities in heavy drinkers was higher (≥ 4 cups per day) than the recent estimates for the hepatoprotective effects of coffee in U.S. National Health and Nutrition Examination Survey (≥ 3 cups per day) Xiao et al. [67] or in the Singapore Chinese Health study (≥ 2 cups per day) [68].

The primary strengths of the current study include the large number of participants, detailed assessments of risk factors, standardized evaluation of alcohol consumption and other risk profiles as well as parallel assessments in men and women. The cross-sectional setting of the survey can be kept as a limitation of this study since lack of follow-up data prevents analyses on the specific relationships between changes in liver enzyme activities and duration of drinking or indices of adiposity. It is also possible that the alcohol recall techniques may lead to underestimation of alcohol intake and overestimate the proportion of those not drinking alcohol at all [29, 69]. This would, however, rather lead to diluting effects to the observed results and the real associations and interactions might be even stronger. It should further be noted that previous studies have emphasized the importance of differentiating between former drinkers, current drinkers and abstainers in studies evaluating health-related effects of alcohol drinking [70]. In our analyses, the individuals who could be classified as former drinkers with no alcohol consumption during the past one year prior to sampling showed low rates of abnormal values underscoring the impact of successful behavioural changes in improving liver health.

Taken together, our study provides a comprehensive assessment of the relationships between alcohol intake and risk of abnormal liver enzyme levels, which may occur even at light to moderate levels of drinking in an age-, gender-, BMI-, and physical activity dependent manner. Current data should be considered in public health recommendations and in the definition of safe limits of ethanol intake.
